# *Borrelia miyamotoi* sensu lato Seroreactivity and Seroprevalence in the Northeastern United States

**DOI:** 10.3201/eid2007.131587

**Published:** 2014-07

**Authors:** Peter J. Krause, Sukanya Narasimhan, Gary P. Wormser, Alan G. Barbour, Alexander E. Platonov, Janna Brancato, Timothy Lepore, Kenneth Dardick, Mark Mamula, Lindsay Rollend, Tanner K. Steeves, Maria Diuk-Wasser, Sahar Usmani-Brown, Phillip Williamson, Denis S. Sarksyan, Erol Fikrig, Durland Fish, the Tick Borne Diseases Group

**Affiliations:** Yale School of Public Health, New Haven, Connecticut, USA (P.J. Krause, J. Brancato, L. Rollend, T.K. Steeves, M. Diuk-Wasser, D. Fish);; Yale School of Medicine, New Haven (P.J. Krause, S. Narasimhan, M. Mamula, E. Fikrig);; New York Medical College, Valhalla, New York, USA (G.P. Wormser);; University of California, Irvine, California, USA (A.G. Barbour);; Central Research Institute of Epidemiology, Moscow, Russia (A.E. Platonov);; Nantucket Cottage Hospital, Nantucket, Massachusetts, USA (T. Lepore);; Mansfield Family Practice, Mansfield, Connecticut, USA (K. Dardick);; L2 Diagnostics, LLC, New Haven (S. Usmani-Brown);; Creative Testing Solutions, Tempe, Arizona, USA (P. Williamson);; State Medical Academy, Izhevsk, Russia (D.S. Sarksyan)

**Keywords:** Borrelia miyamotoi infection, tick-borne disease, ticks, relapsing fever, seroprevalence, seroreactivity, Borrelia miyamotoi sensu lato, United States, northeastern United States, spirochete, bacteria, Borrelia burgdorferi, New York State, New England, Lyme disease

## Abstract

*Borrelia miyamotoi* sensu lato, a relapsing fever *Borrelia* sp., is transmitted by the same ticks that transmit *B. burgdorferi* (the Lyme disease pathogen) and occurs in all Lyme disease�?"endemic areas of the United States. To determine the seroprevalence of IgG against *B. miyamotoi* sensu lato in the northeastern United States and assess whether serum from *B. miyamotoi* sensu lato�?"infected persons is reactive to *B. burgdorferi* antigens, we tested archived serum samples from area residents during 1991�?"2012. Of 639 samples from healthy persons, 25 were positive for *B. miyamotoi* sensu lato and 60 for *B. burgdorferi*. Samples from �%^10% of *B. miyamotoi* sensu lato�?"seropositive persons without a recent history of Lyme disease were seropositive for *B. burgdorferi*. Our resultsA suggest thatA human *B. miyamotoi*A sensu latoA infection may be common in southern New England and that *B. burgdorferi* antibody testing is not an effective surrogate for detecting *B. miyamotoi* sensu lato infection.

## Abstract 2

Serum from �%^4% of residents was positive for infection, compared with �%^9% for *B. burgdorferi*.

Relapsing fever, an arthropod-borne infection caused by several *Borrelia* spp. spirochetes, is transmitted by ticks and lice (*1*,*2*). In 1995, Fukunaga et al. (*3*) discovered a novel relapsing fever spirochete in the hard-bodied (ixodid) tick *Ixodes persulcatus* and named it *Borrelia miyamotoi*. This discovery greatly expanded the potential geographic range of relapsing fever borreliae for humans. Before this finding, only soft-bodied ticks were known to transmit tick-borne relapsing fever spirochetes to humans. In 2001, a related spirochete was detected in *I. scapularis* ticks in the northeastern United States (*4*); this and similar organisms have been designated *B. miyamotoi* sensu lato to distinguish them from the *B. miyamotoi* sensu stricto isolates from Japan (*5*). A subsequent study showed that ticks in 15 states in the northeastern and northern midwestern regions of the United States are infected with *B. miyamotoi* sensu lato and have an average prevalence of infection of 1.9% (range 0�?"10.5%) (*6*). *B. miyamotoi* sensu lato has now been found in all tick species known to be vectors of Lyme disease, including *I. pacificus* in the western United States, *I. ricinus* in Europe, and *I. persulcatus* and *I. ricinus* in Russia (*7*�?"*9*). The first human cases of *B. miyamotoi* sensu lato infection were reported from central Russia in 2011 (*9*). Several reports of *B. miyamotoi* sensu lato infection in humans have subsequently been published, including 3 in the United States, 1 in Europe, and 1 in Russia (*10*�?"*14*). Some of these reports suggest that *B. miyamotoi* sensu lato infection causes a nonspecific, virus-like illness. *B. miyamotoi* sensu lato and *B. burgdorferi*, the agent of Lyme disease, share several antigens that might cause cross-reactivity during serologic testing, which could lead to a misdiagnosis.

There are few data on the seroprevalence of *B. miyamotoi* sensu lato infection. To increase knowledge of the seroprevalence of this infection, we used assays for antibodies against *B. miyamotoi* sensu lato glycerophosphodiester phosphodiesterase (GlpQ), a protein that is absent from all Lyme disease *Borrelia* species (*15*), for evaluation of >1,000 archived serum samples from persons living in a Lyme disease�?"endemic region of the United States. We also performed standard 2-tiered testing for *B. burgdorferi* antibodies (*16*). Our aim was to compare the seroprevalence of *B. miyamotoi* sensu lato with that of *B. burgdorferi*. We also sought to determine whether persons seropositive for *B. miyamotoi* sensu lato would also have positive results for standard *B. burgdorferi* antibody testing.

## Materials and Methods

### Study Population

The serum samples evaluated in our study were obtained during 1991�?"2012 from 3 groups of persons living in areas of the northeastern United States where Lyme disease is endemic. Group 1 consisted of 639 persons from Block Island and Prudence Island, Rhode Island, and from Brimfield, Massachusetts, who participated in serosurveys for tick-borne infections. Persons participating in the serosurvey were healthy at the time of blood sampling and were enrolled during the spring and autumn of each year (*16*). All participants were asked to respond to a questionnaire and to provide a blood sample for serologic analyses of tick-borne infections.

Group 2 consisted of 194 patients from Block Island; Nantucket, Massachusetts; Mansfield, Connecticut; and the Lower Hudson Valley, New York, who were enrolled in studies of tick-borne diseases. At or near the time of sample collection, persons in this group were treated with doxycycline, amoxicillin, or amoxicillin/clavulanic acid for acute Lyme disease.

Group 3 consisted of 221 adult patients who experienced a febrile illness in the late spring or summer without features suggestive of an upper respiratory tract infection or gastroenteritis. A subgroup of group 3 consisted of 17 patients from the Lower Hudson Valley who were enrolled in a study during 1992�?"2009 to better characterize the clinical and laboratory features of human granulocytic anaplasmosis as a single infection or as a co-infection with early Lyme disease. Results for serologic testing, culture, buffy coat examination for morulae, and/or PCR showed that none of the patients was infected with *Anaplasma phagocytophilum* (*17*). All these patients resided in an area where *I. scapularis*�?"transmitted infection is highly endemic and, thus, had possible exposure to *I. scapularis* ticks. A second subgroup of group 3 consisted of 204 adult patients from Block Island, Mansfield, or Nantucket who had suspected Lyme disease or babesiosis. Testing showed that 25 of these patients had babesiosis but none had Lyme disease or anaplasmosis.

Serum samples were also obtained from 2 patients from the Udmurtia Republic, Russia, who had PCR-confirmed acute *B. miyamotoi* sensu lato infection. In addition, Creative Testing Solutions (Tempe, AZ, USA) provided an aliquot of residual serum used for blood screening from 300 blood donors who lived in Tempe or in Miami, Florida.

De-identified serum samples were used in this study. The study was approved by the Yale School of Public Health Human Investigation Committee, the New York Medical College Institutional Review Board, and the University of Connecticut Institutional Review Board.

### Laboratory Procedures

#### Production of *B. miyamotoi* sensu lato GlpQ Antigen

**Figure 1:**
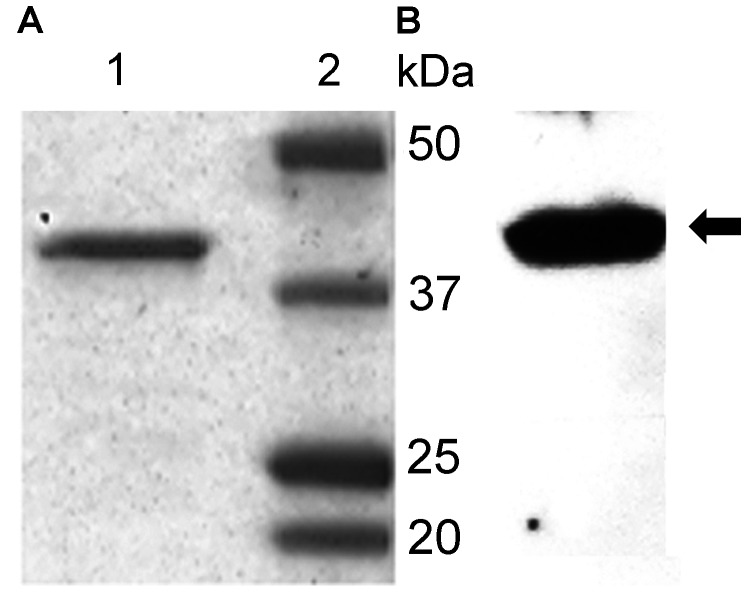
Polyacrylamide gel electrophoresis purification (A) and Western blot analysis (B) of recombinant glycerophosphodiester phosphodiesterase (rGlpQ). A) Coomassie blue staining of purified *Borrelia miyamotoi* sensu lato rGlpQ (lane 1) and of Precision Plus Protein Prestained Standards (Bio-Rad, Laboratories, Hercules, CA, USA) (lane 2). B) Western blot analysis of *B. miyamotoi* sensu lato�?"positive control mouse serum shows 39-kDa rGlpQ-specific band (arrow).

*B. miyamotoi* sensu lato *glpQ* from strain LB-2001 cloned into the prokaryotic expression vector pXT7 (*18*), a derivative of pGEM4Z and pSP64T (Promega, Madison, WI, USA), was transformed into BL21 Star (DE3)/pLysS cells (Invitrogen, Carlsbad, CA, USA), and transformants were used for protein production (*6*). The chromosome sequence for the protein is in GenBank (accession no. CP006647) (*19*). The 39.1-kDa recombinant GlpQ (rGlpQ) containing an N-terminal His tag was purified over an Ni-NTA Superflow affinity column (QIAGEN, Valencia, CA, USA) as described by the manufacturer. Purity was assessed by sodium dodecyl sulfate electrophoresis of �%^1 I1/4g of rGlpQ on a 4%�?"20% polyacrylamide gel and by Coomassie blue staining (Figure 1).

#### GlpQ Antibody ELISA

We developed a *B. miyamotoi* sensu lato IgG ELISA by using 20 C3H/HeJ mice (Jackson Laboratory, Bar Harbor, ME, USA). Ten of the mice were not infected. The other 10 age-matched mice were infected by using *B. miyamotoi* sensu lato�?"infected *I. scapularis* nymphal ticks. A month after the mice were infected, blood was obtained from all 20 mice for testing. Titrating concentrations of GlpQ protein and secondary antibody were tested in a checkerboard assay to determine the optimal concentrations for detecting *B. miyamotoi* sensu lato antibody. Results for the *B. miyamotoi* sensu lato ELISA were positive for all 10 *B. miyamotoi* sensu lato�?"infected mice and negative for all 10 uninfected mice.

To test the human serum samples, we coated ELISA plates with 100 I1/4L of 1 I1/4g/mL GlpQ protein in phosphate-buffered saline (PBS) and incubated the plates at 4A�C for 18 h. We then added 300 I1/4L of 1% bovine serum albumin in PBS buffer to the plates and incubated them for 2 h at room temperature. The plates were then emptied, and serum was added at a 1:320 dilution and incubated for 1 h. If acute- and convalescent-phase serum samples were available for a study participant, the initial dilution of the acute-phase sample was 1:80, and convalescent-phase samples were diluted to endpoint. The plates were then washed 3 times with wash buffer, and 100 I1/4L of goat antihuman IgG secondary antibody was added at 0.002 mg/mL, incubated for 1 h, and then washed 3 times. BluPhos substrate (Kirkegaard & Perry, Gaithersburg, MD, USA) was added and allowed to react for 20 min before absorbance at 630 nm was determined. *B. miyamotoi* sensu lato�?"infected mouse serum was used as a positive control. As a negative control for each plate, we used serum samples that were negative for *B. miyamotoi* sensu lato antibody, as determined by ELISA and Western blot. The serum was obtained from 3 healthy participants who had no history of tick bite or tick-borne disease and who lived in an area where Lyme disease is endemic. The serum samples were tested by PCR for amplifiable *B. miyamotoi* sensu lato DNA and were negative. For mouse and human serum samples, a signal >3 SD above the mean of 3 noninfected serum controls was considered positive for *B. miyamotoi* sensu lato infection.

#### GlpQ Western Blot Antibody Assay

Purified GlpQ (500 ng) was electrophoresed on each replicate lane of a precast mini 4%�?"20% sodium dodecyl sulfate�?"polyacrylamide gel electrophoresis gel (Bio-Rad Laboratories, Hercules, CA, USA) and transferred to a nitrocellulose membrane using the Bio-Rad MiniTrans Blot Cell (Bio-Rad Laboratories). Replicate strips containing rGlpQ were blocked overnight at 4A�C in PBS (pH 7.2)/5% dried milk/0.05% Tween 20. The blocked strips were then individually incubated with human serum at a 1:250 dilution at room temperature in PBS (pH 7.2)/2.5% dried milk/0.05% Tween 20 for 1 h. The strips were then washed 3 times and incubated for 1 h with horseradish peroxidase�?"conjugated rabbit anti-human IgG (Sigma-Aldrich, St. Louis, MO, USA) or with horseradish peroxidase�?"conjugated goat anti-human IgM (Invitrogen) at a 1:5,000 dilution in PBS (pH 7.2)/2.5% dried milk/0.05% Tween 20. Bound antibodies were detected by using Thermo Scientific SuperSignal West Pico Chemiluminescent Substrate (Thermo Fisher Scientific, Inc., Rockford, IL, USA). Serum from �%^10% of the study participants reacted to a �%^55-kDa band, presumably a trace contaminant copurified with the rGlpQ generated in a bacterial expression system. Samples with a 39-kDa band corresponding to GlpQ on positive control mouse serum samples were considered GlpQ antibody�?"positive (Figure 1).

#### PCR DNA Amplification

We used a *B. miyamotoi* sensu lato PCR as described (*4*) to amplify *B. miyamotoi* sensu lato DNA in serum samples. *B. burgdorferi* DNA was amplified by using a standard PCR assay (*16*).

#### *B. burgdorferi* Antibody Detection

We detected serologic evidence of exposure to *B. burgdorferi* by using a whole-cell sonicate ELISA, C6 ELISA, or Western blot assay as described (*16*,*20*�?"*22*). Specimens were considered positive according to the criteria of the US Centers for Disease Control and Prevention (http://www.cdc.gov/lyme/diagnosistesting/LabTest/TwoStep/index.html).

### Case Definitions

*B. miyamotoi* sensu lato�?"seropositive serum samples were defined by the presence of *B. miyamotoi* sensu lato antibody as determined by using ELISA and confirmatory Western blot assays for IgG alone or IgG plus IgM antibody. *B. burgdorferi* seropositive serum samples were defined by the presence of *B. burgdorferi* antibody as determined by ELISA and supplemental Western blot IgM or IgG assays.

Study participants were considered to have *B. miyamotoi* sensu lato infection if they had exhibited a fever >37.5A�C and a >4-fold rise in antibody to *B. miyamotoi* sensu lato GlpQ protein between acute- and convalescent-phase serum samples, as determined by ELISA and confirmatory Western blot assays for IgG alone or IgG plus IgM. The time between acute- and convalescent-phase samples ranged from 2 wk to 2 mo. Study participants were considered to have Lyme disease if they had a physician-diagnosed erythema migrans skin lesion or a virus-like illness plus a test result that showed either PCR amplification of *B. burgdorferi* DNA in blood or *B. burgdorferi* seroconversion from negative to positive between acute- and convalescent-phase serum samples.

### Statistical Analysis

A 2-tailed Fisher exact test was used to compare the frequency of *B. miyamotoi* sensu lato�?"seropositive and �?"seronegative study participants in groups 1, 2, and 3. The McNemar I�^2^ test was used to compare the seroprevalence of *B. miyamotoi* sensu lato and *B. burgdorferi* among group 1 participants.

## Results

### Seroprevalence of *B. miyamotoi* sensu lato Infection

Serum samples from 52 of the 1,054 study participants were seroreactive to *B. miyamotoi* sensu lato antigen by rGlpQ ELISA and Western blot assay (Table 1). The percentage of *B. miyamotoi* sensu lato�?"seropositive persons was greater among participants with Lyme disease (group 2; 19/194 [9.8%]) than among those who were healthy (group 1; 25/639 [3.9%], p<0.01 by Fisher exact test, odds ratio [OR] 2.66 [range 1.35�?"5.16]) or those who had a febrile illness in the late spring or summer (group 3; 8/221 [3.6%], p<0.05 by Fisher exact test, OR 2.89 [range 1.17�?"7.81]). *B. miyamotoi* sensu lato DNA could not be amplified from any serum samples (including 27 acute-phase serum samples) from the 52 participants who had test results positive for *B. miyamotoi* sensu lato antibody.

**Table 1:** Assay results for patient samples seroreactive to *Borrelia miyamotoi* sensu lato antigen, northeastern United States, 1991�?"2012

Group no, description, participant no.	Year sample obtained	IgG ELISA	Western blot IgM	Western blot IgG
Group 1, healthy participants, n = 639				
1	1995	1:320	Positive	Positive
2	2000	>1:1280	Negative	Positive
3	1991	>1:1280	Positive	Positive
4	1993	>1:1280	Negative	Positive
5	2000	>1:1280	Negative	Positive
6	2000	>1:1280	Negative	Positive
7	2012	>1:1280	Negative	Positive
8	2012	>1:1280	Negative	Positive
9	2012	>1:1280	Negative	Positive
10	2012	>1:1280	Negative	Positive
11	1993	>1:1280	Negative	Positive
12	1993	1:320	Negative	Positive
13	2012	1:320	Negative	Positive
14	2012	>1:1280	Positive	Positive
15	2012	1:640	Negative	Positive
16	2002	>1:1280	Negative	Positive
17	2002	>1:1280	Negative	Positive
18	2002	>1:1280	Negative	Positive
19	2002	>1:1280	Negative	Positive
20	2000	>1:1280	Negative	Positive
21	2000	>1:1280	Negative	Positive
22	2002	>1:1280	Negative	Positive
23	2002	>1:1280	Negative	Positive
24	2002	>1:1280	Positive	Positive
25	2002	>1:1280	Positive	Positive
Group 2, adults with Lyme disease, n = 194				
26				
Acute-phase serum	1992 Jul 17	1:80 (negative)	Negative	Negative
Convalescent-phase serum	1992 Jul 27	1:1280	Negative	Positive
27				
Acute-phase serum	1997 Jul 27	1:160 (negative)	Negative	Negative
Convalescent-phase serum	1997 Aug 26	1:1280	Positive	Positive
28				
Acute-phase serum	1996 Jun 30	1:80 (negative)	Positive	Positive
Convalescent-phase serum	1996 Jul 10	1:320	Negative	Positive
29				
Acute-phase serum	1997 Aug 7	1:80 (negative)	Negative	Negative
Convalescent-phase serum	1997 Aug 17	>1:1280	Negative	Positive
30	1995	>1:1280	Negative	Positive
31	1991	>1:1280	Positive	Positive
32	2004	1:640	Negative	Positive
33	2004	1:320	Positive	Positive
34	2000	>1:1280	Negative	Positive
35	2011	>1:1280	Negative	Positive
36	1995	>1:1280	Negative	Positive
37	1994	>1:1280	Negative	Positive
38	1998	1:320	Positive	Positive
39	2000	>1:1280	Negative	Positive
40	1998	>1:1280	Positive	Positive
41	2006	>1:1280	Negative	Positive
42	2002	>1:1280	Negative	Positive
43	2002	1:320	Negative	Positive
44	1995	1:320	Positive	Positive
Group 3, adults with virus-like illness, n = 221				
45				
Acute-phase serum	1996 Jul 8	1:80 (negative)	Positive	Negative
Convalescent-phase serum	1996 Jul 19	1:320	Positive	Positive
46	2011	>1:1280	Positive	Positive
47	1997	>1:1280	Negative	Positive
48	1991	>1:1280	Negative	Positive
49	1991	>1:1280	Negative	Positive
50	1993	>1:1280	Negative	Positive
51	1997	1:320	Negative	Positive
52	1992	1:320	Negative	Positive

Of the 639 serum samples from group 1 participants, 25 (3.9%) were seroreactive to *B. miyamotoi* sensu lato antigen and 60 (9.4%) were seroreactive to *B. burgdorferi* antigen, as determined by using the standard 2-step ELISA and Western blot procedure (McNemar I�^2^ test, p<0.0001, OR 10.23 [range 7.84�?"13.57]). About half (51%) of group 1 participants were male, and the mean age of group 1 participants was 55 years (range 2�?"102). There was no significant difference in the sex of the group 1 study participants who were seropositive for *B. miyamotoi* sensu lato (40% male) and those who were seropositive for *B. burgdorferi* (53% male; p = 0.34). The mean age also did not differ significantly between participants who were seropositive for *B. miyamotoi* sensu lato (59 years [+15]) and those who were seropositive for *B. burgdorferi* (61 years [+15]; p = 0.62).

Of the participants from Brimfield, Massachusetts, 9.3% (10/107) were seropositive for *B. miyamotoi* sensu lato and 7.5% (8/107) were seropositive for *B. burgdorferi*, compared with 3.2% (15/474) and 11% (52/474), respectively, of the participants from Block Island, Rhode Island. None of the 58 participants from Prudence Island, Rhode Island, were seropositive for *B. miyamotoi* sensu lato or *B. burgdorferi*.

### Serodiagnosis of *B. miyamotoi* sensu lato Infection

To assess *B. miyamotoi* sensu lato ELISA and Western blot assay accuracy in patients with confirmed *B. miyamotoi* infection, we tested acute- and convalescent-phase serum samples from 2 patients in Russia with *B. miyamotoi* sensu lato infection confirmed by real-time PCR�?"(*9*). Both patients had a >4-fold rise in *B. miyamotoi* sensu lato GlpQ antibody between acute- and convalescent-phase serum samples (1:80 and 1:2,560, respectively, for 1 patient and 1:640 and 1:2,560, respectively, for the other), as determined by ELISA and confirmed by Western blot.

To assess *B. miyamotoi* sensu lato ELISA and Western blot assay performance in persons at low risk for Lyme disease or *B. miyamotoi* sensu lato infection, we performed the GlpQ ELISA on 300 serum samples from healthy blood donors living in Tempe or Miami. For the 9 microtiter plates used for this serosurvey, the mean and standard deviation of the ELISA optical density values for 3 negative control serum samples ranged from 0.108 to 0.136 and from 0.03 to 0.07, respectively. Of the 300 samples, 19 (6.3%) exceeded the mean of the negative control serum by >3 SDs, but none was reactive by Western blot.

We determined whether *B. miyamotoi* sensu lato infection might be misdiagnosed as Lyme disease in persons whose serum was reactive by *B. burgdorferi* antibody testing. Of the 36 *B. miyamotoi* sensu lato�?"seropositive study participants without a clinical history of Lyme disease within the previous 2 years, 7 (19.4%) had test results positive for *B. burgdorferi* by IgG and/or IgM ELISA, 6 (16.7%) had test results positive for C6 ELISA, and 4 (11.1%) had test results positive for standard 2-tier ELISA plus confirmatory Western blot (Table 2). The 2-tier *B. burgdorferi* ELISA and Western blot assay combination used in our laboratory has a 2% false-positive rate.

**Table 2:** Number of false-positive *Borrelia burgdorferi* assay results for participants in various relapsing fever studies*

*Borrelia burgdorferi* assay	No. participants seroreactive to *B. burgdorferi*/no. total (%)				
	*B. miyamotoi* (current study)�?	*B. hermsii* (*23*)	*B. hermsii* (*24*)	*B. recurrentis* (*24*)	*B. recurrentis* (*25*)
Whole-cell sonicate ELISA	7/36 (19)	ND	7/11 (64)	3/11 (27)	5�?"7/11 (45�?"64)
C6 ELISA	6/35 (17)�?�	1/14 (7)	ND	ND	ND
Whole-cell sonicate ELISA and Western blot	4/36 (11)	2/14 (14)	ND	ND	ND

*ND, not determined.�?"�? Study participants had no history of Lyme disease within the 2 years before serum was obtained for testing.�?"�?�The quantity of 1 serum sample was insufficient to test.

### Clinical Manifestations among Patients with *B. miyamotoi* sensu lato Seroconversion

**Figure 2:**
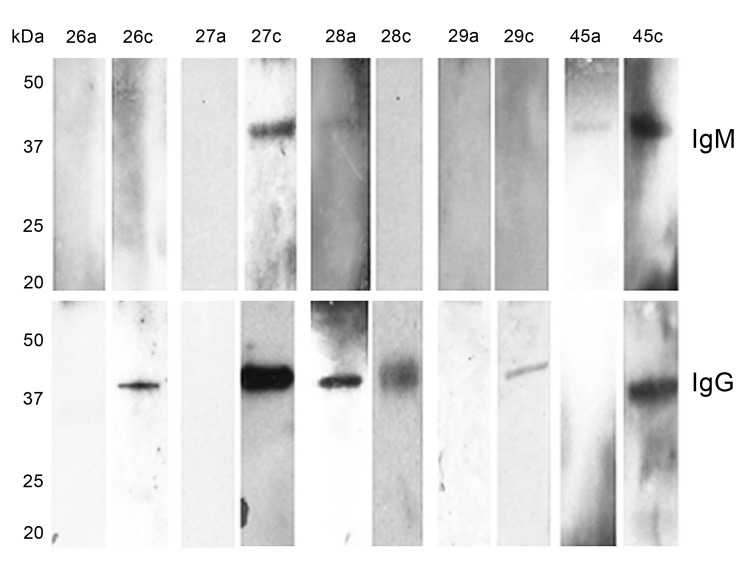
Western blot reactivity to recombinant *Borrelia miyamotoi* glycerophosphodiester phosphodiesterase in serum samples from 5 *Borrelia miyamotoi* sensu lato�?"seropositive patients in the northeastern United States, 1991�?"2012. Numbers at the top of rows are patient numbers and correspond to patients 26-29 and 45 in Table 1. The letters a and c that follow patient numbers indicate acute- and convalescent-phase serum samples, respectively. Western blot results that show no seroreactive IgG and/or IgM band in acute-phase serum samples and a reactive IgG and/or IgM band in convalescent-phase serum samples are consistent with ELISA results showing a 4-fold rise in *B. miyamotoi* sensu lato antibody titer from acute-phase (negative) and convalescent-phase (positive) serum samples. The acute-phase serum of patient 28 was nonreactive for IgG in the ELISA assay (Table 1), but the sample was reactive for IgM and IgG on Western blot.

A clinical description of illness was available for 5 symptomatic patients who experienced a �%�4 fold rise in *B. miyamotoi* sensu lato IgG and/or IgM antibody between acute- and convalescent-phase serum samples, as determined by ELISA and confirmatory Western blot assays (Western blot data shown in Figure 2). Of the 5 patients, 4 were co-infected with Lyme disease, 1 of whom was also co-infected with babesiosis (determined by blood smear). The 4 patients all had an erythema migrans skin lesion, and 2 had culture results positive for *B. burgdorferi*. The fifth patient had no evidence of co-infection and was the only 1 of 17 (5.9%) participants with a febrile summertime illness who had acute- and convalescent-phase serum tested for *B. miyamotoi* sensu lato antibody and who seroconverted. Three of these 5 patients have been reported previously (*11*). All 5 patients had fever, but a relapsing fever pattern was not reported. Symptoms resolved in 4 of the patients after treatment with doxycycline for 7�?"14 days, and symptoms resolved in the fifth patient after treatment with amoxicillin/clavulanic acid for 14 days.

## Discussion

We found evidence of human infection with the spirochete *B. miyamotoi* sensu lato in 52 residents residing in southern New England or New York State during 1991�?"2012. Among healthy study participants from southern New England, the seroprevalence of *B. miyamotoi* sensu lato infection was about one third that of *B. burgdorferi* infection (3.9% vs. 9.4%, respectively). This finding is consistent with the higher rate of *B. burgdorferi* infection in *I. scapularis* ticks in the region (range 2:1�?"20:1) (*4*�?"*6*). As expected, the seroprevalence of *B. miyamotoi* sensu lato infection was higher in serum samples from patients with acute Lyme disease and recent *I. scapularis* tick bites than in serum samples from patients whose tick-bite status was unclear. *B. miyamotoi* sensu lato seroprevalence rates were similar among study participants with a febrile late spring or summertime illness and healthy participants, probably because *B. miyamotoi* sensu lato infection is unlikely to be a common cause of nonspecific febrile illness in the late spring or summer. The seroprevalence of *B. miyamotoi* sensu lato was less than that of *B. burgdorferi* but similar to that of *Babesia microti* among residents of the same southern New England and New York region (*16*,*26*�?"*28*).

Approximately 10 percent of the *B. miyamotoi* sensu lato�?"seropositive patients without a recent history of Lyme disease reacted to *B. burgdorferi* antigen by 2-tier testing. The reactivity could have represented a prior *B. burgdorferi* infection, a false-positive test reaction, and/or cross-reactivity of *B. miyamotoi* sensu lato antibody against >1 *B. burgdorferi* antigens. The frequency of antibody reactivity to *B. burgdorferi* in patients with relapsing fever is shown in Table 2 (*23*�?"*25*,*29*). Several proteins are found in common between *B. burgdorferi* and *B. miyamotoi* sensu lato, including the flagellin FlaB protein, the GroEL heat shock proteins, and the BmpA (P39) protein (*19*,*25*). Misdiagnosis of *B. miyamotoi* sensu lato infection as Lyme disease is therefore possible. Results of *B. burgdorferi* testing may be positive for *B. miyamotoi* sensu lato�?"infected patients who are co-infected with *B. burgdorferi* (as was the case for some persons in this study). Our findings suggest, however, that testing for antibodies against *B. burgdorferi* is not an appropriate surrogate for testing for antibodies against *B. miyamotoi* sensu lato; *B. burgdorferi* antibody testing should not be used in place of an assay for antibody against *B. miyamotoi* sensu lato GlpQ or another *B. miyamotoi* sensu lato�?"specific antigen.

Our study had several limitations. First, laboratory evidence for acute *B. miyamotoi* sensu lato infection was based on ELISA and Western blot antibody assay rather than on culture, blood smear, or *B. miyamotoi* sensu lato PCR. However, in agreement with the case definition commonly used for many infectious diseases by the US Centers for Disease Control and Prevention (*30*), we considered results positive if a >4-fold rise in antibody occurred between acute- and convalescent-phase serum samples.

Second, *B. miyamotoi* sensu lato from North America has not been cultured, and blood smears were not available from the patients in our study. We were unable to detect *B. miyamotoi* sensu lato DNA in frozen, archived serum samples; however, the process of preparing serum from whole blood likely removed some spirochetes from the samples, and freeze�?"thaw cycles may have destroyed bacterial DNA. Furthermore, almost half of the serum samples that we tested were obtained after the period of acute illness, when the bacteremia may have cleared.

Third, our seroprevalence rates presumably would have been higher if we had tested for both IgM and IgG antibody by ELISA and included patients with IgM antibody alone as seropositive patients; however, we chose a more stringent definition of seropositivity by requiring the presence of IgG antibody. On the other hand, our seroprevalence data may have been inflated as a result of cross-reactivity of antibodies from other infections reacting against *B. miyamotoi* sensu lato GlpQ antigen. Although all other relapsing fever species have the *glpQ* gene, no other relapsing fever *Borrelia* sp. has been identified in *I. scapularis* ticks or humans in the northeastern United States (*2*,*4*�?"*6*,*15*,*17*,*31*).

Last, we do not have travel histories for the *B. miyamotoi* sensu lato�?"seroreactive patients included in the study, but the probability that many of our patients would have had exposure to other relapsing fever *Borrelia* spp. in the United States seems highly unlikely because these infections are infrequent and occur in the western states (*2*). Cross-reactivity against other tick-borne infections in the Northeast also appears unlikely because the agents of Lyme disease, human granulocytic anaplasmosis, and Powassan virus disease lack a *glpQ* gene (*15*). Proteins homologous to the GlpQ protein of relapsing fever borreliae are found in some gram-negative bacteria, including *Escherichia coli*, but they are so distant in sequence that antibody cross-reactivity is not expected (*15*).

The determination of *B. miyamotoi* sensu lato seroprevalence in our population is important because it indicates that this pathogen may infect persons at a rate that is similar to that of *B. microti* in the northeastern United States (*16*,*23*,*24*). Our data suggest that acute *B. miyamotoi* sensu lato infection in some persons may be misdiagnosed as Lyme disease because of the presence of antibody to *B. burgdorferi* from a previous *B. burgdorferi* infection, a false-positive test reaction, and/or cross-reactivity. Antibody testing for *B. burgdorferi*, however, is not adequate to detect infection with *B. miyamotoi* sensu lato in the United States. The potential for misdiagnosis may be greater in locations like northern California, were the prevalence of *B. miyamotoi* sensu lato in ticks equals or exceeds the prevalence of *B. burgdorferi* in ticks (*32*). Further studies are needed to better characterize the epidemiology and improve the serodiagnosis of human *B. miyamotoi* sensu lato infection.
